# Transformative blockchain technological approaches to sports events

**DOI:** 10.3389/fspor.2025.1547137

**Published:** 2025-06-16

**Authors:** Vitor Principe, Tiago Ribeiro, Samuel López-Carril

**Affiliations:** ^1^ Faculty of Sport, University of Porto, Porto, Portugal; ^2^ Research Center in Sports Sciences, Health Sciences and Human Development (CIDESD), University of Maia, Maia, Portugal; ^3^ Laboratory of Exercise and Sports (LABEES), Rio de Janeiro State University (UERJ), Rio de Janeiro, Brazil; ^4^ Faculty of Human Kinetics, University of Lisbon, Lisbon, Portugal; ^5^ Research Center for Tourism, Sustainability, and Wellbeing (CinTurs), University of Algarve, Faro, Portugal; ^6^ Departamento de Educación Física y Deportiva, Facultad de Ciencias de la Actividad Física y el Deporte, Universitat de València, Valencia, Spain

**Keywords:** decentralized systems, stakeholder systems, organizational innovations, smart infrastructure, digital event management

## Abstract

The integration of blockchain technology in sports event management represents a significant shift towards more decentralized and efficient governance structures, particularly relevant to small and medium-sized events. Despite growing interest, its practical implementation remains limited and lacks comprehensive theoretical guidance. This study addresses this gap by proposing an integrated theoretical framework, combining the Dynamic Capabilities Framework (DCF), Collaborative Governance Theory (CGT), and the Four Modes of Governance (FMG), to systematically explore blockchain's application within sports event management. Our analysis reveals that blockchain technology can effectively foster transparency, efficiency, and enhanced stakeholder participation through Decentralized Autonomous Organizations (DAOs). These advantages are realized through key mechanisms of access, control, and incentives, which interact across external environments, governance structures, and blockchain core infrastructure. Furthermore, the study identifies critical managerial implications necessary for successful blockchain implementation, emphasizing strategic infrastructure assessments, stakeholder engagement, and risk management protocols. Ultimately, this research contributes both theoretical insights and practical guidelines, addressing existing knowledge gaps and providing a structured framework for leveraging blockchain in managing small to mediumsized sports events.

## 1 Introduction

Technological development has been one of the primary drivers of competitiveness in the sports industry, with the intersection of technology, governance, and sustainability reshaping the fundamental principles of management and organization (1). Using technologies enhances athlete performance and transforms the management and operationalization of sports events, providing greater efficiency and effectiveness in organizing and conducting these events (2). In this context, blockchain technology emerges as an innovative architecture that establishes a new “architecture of trust”, enabling multiple actors who do not know (or trust) each other to interact safely under predetermined conditions (3). This is particularly relevant for sports event governance where the technology can facilitate three key governance mechanisms: access, control, and incentives (4). Access mechanisms define who can participate in the platform ecosystem and under which conditions, while control specifies the rules by which potentially competing actors interact, and incentives motivate participation and specific actions of different actors, thus facilitating innovative value creation (5).

These governance mechanisms are inherent to the technical architecture of the platform and thus set the rules of interaction among all actors, addressing the fundamental challenge of enabling maximum openness while ensuring effective value capturing for all participants (4, 6). Applying advanced technologies and analytics allows organizers to understand the target group's needs and market trends, enabling them to operate more efficiently and effectively in future event challenges (7). Additionally, these technologies can contribute to event risk assessment and security management, ensuring that events proceed smoothly and safely (8).

Digital transformation has significantly reshaped how sports events are experienced and managed. Technologies such as virtual reality (VR), augmented reality (AR), and live streaming offer immersive and personalized experiences, allowing spectators to feel part of the event remotely (9–11). VR applications in events like the Paris 2024 Paralympic Games also demonstrate its potential to promote social inclusion (12). In parallel, digital platforms and social media, including OTT services and fantasy league apps, have become essential tools for engagement and real-time communication (7, 13–15). The COVID-19 pandemic accelerated these trends, as virtual and hybrid event formats emerged to maintain athlete activity and fan involvement (16–20). These developments call for innovative governance structures that ensure transparency and stakeholder participation in increasingly digital sports ecosystems.

Notwithstanding the significant technological advancements and escalating adoption of blockchain technology in sports events (21, 22), a substantial lacuna persists in the implementation of Decentralized Autonomous Organizations (DAOs) for event governance frameworks (23). While DAOs offer transparency and participatory decision-making (24), their implementation in sports is hindered by regulatory, legal, and technological complexities (25, 26). Contemporary governance models struggle to adapt to the digital and decentralized nature of modern sports events, underscoring the imperative for innovative governance frameworks capable of effectively synthesizing technological advancement with stakeholder interests (27).

This research endeavour addresses these gaps identified by proposing a comprehensive theoretical framework that synthesizes DAOs with established governance theories, thereby advancing event management efficacy and stakeholder participation. The design of such a framework must consider both the endogenous and exogenous legitimacy of blockchain systems (6). Endogenous legitimacy emerges from the network's internal mechanisms, consensus protocols, and adherence to predefined rules, while exogenous legitimacy stems from interactions with actors outside the blockchain ecosystem, including regulatory compliance and legal recognition. This dual approach to legitimacy is particularly crucial for sports events where governance systems must balance internal operational efficiency with external stakeholder expectations and regulatory requirements (4). The study's empirical investigation is guided by the following research inquiries:

1. To what extent can DAOs facilitate the transformation of governance architectures within small and medium-sized sports events to enhance stakeholder engagement and operational efficacy?
2. How can the theoretical integration of the dynamic capabilities framework with governance theories provide a conceptual framework for implementing DAOs in small and medium-sized sports events?

Theoretically, this study will contribute to conceptualizing the role of decentralized organizations in managing small and medium-sized sports events. By using a strategic management lens for differentiation, the dynamic capabilities framework (DCF) together with two governance theoretical lenses—the collaborative governance theory (CGT) and four modes of governance (FMG) in using blockchain technology—we aim to demonstrate how the development of DAOs can be instrumental in fostering open participation, direct interactions, and community decision-making in sports events. This approach emphasizes that continuous adaptation and transformation capabilities are essential for sports organizations to thrive in dynamic and rapidly changing environments.

### 1.1 Objective and structure

This study possesses a distinctly theoretical and conceptual objective to (a) investigate the application of blockchain technologies in managing small and medium-scale sports events with a focus on decentralization and operational efficiency, and (b) examine the synergistic application of the Four Modes of Governance and Collaborative Governance frameworks, utilizing blockchain technology to enhance the management of sports events. Consequently, the primary contributions of this study include forming a theoretical model that integrates these governance frameworks with blockchain technology, thereby offering a novel perspective on decentralized governance and community engagement in sports events. Furthermore, the study elucidates the theoretical and practical implications of these integrations, proposing methods by which sports and social values can be enhanced through emerging technologies.

The integration of blockchain technology in sports event management represents a transformative opportunity. However, this subject requires caution, as existing research on the use of technology still presents significant gaps. There is a distinct lack of empirical evidence demonstrating practical outcomes of blockchain implementation within real-world sports events (28). Practical implementation examples remain limited, leaving uncertainty around blockchain's effectiveness in actual event management scenarios. Also, essential barriers have been inadequately addressed in prior studies. Key issues, such as technological literacy among stakeholders, governance disputes arising from decentralized decision-making, and resistance to technology adoption are either overlooked or insufficiently explored in the literature (e.g., the lack of standardization and interoperability between blockchain systems in sports event management, regulatory uncertainties affecting compliance with digital asset laws, and the scalability limitations of blockchain networks for real-time ticketing and transactions in major events).

The conceptualization of blockchain applications in event management remains underdeveloped, with theoretical frameworks struggling to capture its decentralized, automated, and multi-stakeholder governance potential. Existing literature lacks cohesive models that integrate blockchain's technical mechanisms with event management practices, leading to gaps in understanding its practical implementation, stakeholder dynamics, and governance structures. This study addresses these critical gaps by proposing a comprehensive theoretical framework that integrates the Dynamic Capabilities Framework (DCF), Collaborative Governance Theory (CGT), and the Four Modes of Governance (FMG). Our proposed framework guides overcoming barriers related to governance disputes, technological literacy challenges, and stakeholder resistance. It also incorporates regulatory and legal considerations essential for practical implementation, thereby advancing theoretical understanding and managerial practices in sports event management.

The article is systematically organized into six principal sections. Following this introduction, the subsequent section addresses the theoretical foundation, elaborating on the role of blockchain, the FMG, and CGT within the context of sports events. The methodology section, predominantly conceptual, delineates the theoretical framework and proposes a series of reflections grounded in literature reviews and deductive analyses. Anticipated results are discussed in sequence concentrating on governance theories and technological innovation in sports events management. The conclusion encompasses a comprehensive discussion regarding the theoretical implications of the results, offering managerial insights for the sports events industry and the domain of technological management. Lastly, we acknowledge the limitations of the study and propose avenues for future research in the technological and event management field.

## 2 Literature review

### 2.1 Technological applications in sporting events

Technology has become one of the most critical factors driving competitiveness in the sports industry. It can be understood as applying scientific knowledge to create tools and systems that solve practical problems (2). When applied to sports, technology goes beyond simply enhancing athlete performance; it encompasses the management and operationalization of sports events, proving essential for the efficiency and effectiveness of such event organizations (7).

In sports event management, technology serves various purposes such as data analysis for strategic decision-making and improving spectator experiences (29). Big data and advanced analytics enable organizers to better understand audience needs and market trends, enabling more targeted and efficient promotion (7). Additionally, these technologies facilitate risk assessment and security management, ensuring that events proceed smoothly and safely (8).

Digitalization has also transformed how sports events are done. Social media platforms and digital marketing have become vital for direct communication with the audience, allowing for closer and more engaging interactions. These platforms disseminate information about events, interactive activities, and evaluations, thus increasing public participation and engagement (7). For instance, the digital transformation in sports events is marked by using advanced surveillance technologies to enhance security, as seen in international stadiums that monitor and manage crowd flow in real time (30, 31). Services for event attendees are personalized through digital platforms, catering to the specific needs of ticket holders (32). Digital twin technology provides fans with an immersive experience and enhances logistical efficiency (33). Additionally, the spectator experience is enriched through virtual reality (VR) and augmented reality (AR) (34). These innovations improve sports management and intensify fan engagement, redefining the sports industry in the digital era. VR, AR, and live streaming have provided more immersive and personalized viewing experiences. As an illustration, fans can feel part of the event even from a distance, choosing different angles and modes of interaction (11). Live streaming also allows games to be watched in real time by a global audience, expanding the reach and impact of sports events (15). Furthermore, integrating electronic sports (eSports) with traditional sports events is another emerging digital event trend. This collaboration attracts young people and opens new sponsorship and innovation opportunities (35). Blending eSports data and broadcasting technologies enriches competition management and spectator experience, creating new modes of competition and interaction (36).

Likewise, the COVID-19 pandemic accelerated the adoption of digital technologies in sports events. The need for social distancing and mobility restrictions led event managers to seek digital solutions to keep events operational (20). Virtual events, eSports, and exergames emerged as viable alternatives, allowing athletes to remain active and fans to stay engaged (16). The virtualization of sports events entertained several target groups and created new revenue and commercial engagement opportunities, benefiting athletes, organizers, and sponsors (19). For example, virtual meet-and-greets, such as those promoted by the NFL, enabled fans to interact with players in a personalized way, while digital event platforms offered live interaction tools, fostering continuous engagement (13). The growing popularity of fantasy sports and co-participation platforms has also transformed the fan experience, allowing them to engage in virtual games and real-time discussions and access exclusive information about their favourite teams and players (37). Co-viewing practices, which surged in popularity, provided fans with real-time social engagement, satisfying needs for entertainment and social integration while increasing their connection to events in ways that transcend traditional broadcasting models (14). These digital initiatives have created new opportunities for engagement and revenue, bringing audiences closer to events and increasing the perceived value of digital sports interactions (35).

An emerging technology that has stood out in sports event management is blockchain. It offers a decentralized and secure solution for multiple challenges sports event managers face (21, 38, 39). Blockchain makes it possible to create immutable and transparent transaction records, increasing trust and security in ticket sales, athlete data management, and copyright protection (22). For instance, nonfungible tokens (NFTs), a technology based on blockchain, have changed sports events management, such as ticket speculation and fan engagement. NFTs can be used to create unique digital tickets that are difficult to counterfeit and easy to track (21, 22). This can improve security and efficiency in ticket sales, offering new paths of interaction and rewards for fans such as loyalty programs and access to exclusive content (21). Furthermore, blockchain is also used to protect the copyrights of sports events, ensuring that digital content is distributed relatively and traceably (40). It allows the creation of immutable records that guarantee the authenticity and ownership of content, reducing piracy and unauthorized distribution of materials related to sports events (28).

In summary, integrating blockchain technology in sports event management makes it possible to promote the transformation and development of new business models and improve operational efficiency and customer experience. In a post-pandemic environment where adaptation and resilience are essential, blockchain offers a robust solution to address challenges and seize emerging opportunities in the sports sector (22, 33). In our study, we will explore transformative blockchain technology approaches within the management of sports events, underpinned by a theoretical-conceptual approach derived from the pertinent literature. We focus on blockchain's decentralization, immutability, and transparency to propose innovative management practices. Drawing on the DCF (41, 42), we emphasize the relevance of an organization's ability to adapt, integrate, and reconfigure internal and external competencies to achieve and sustain competitive advantages. Additionally, we apply the CGT (43) to explore the dynamics of inter-organizational collaboration for effective governance while leverage the FMG (44) framework to illustrate how various governance structures can be operationalized by using blockchain technology.

### 2.2 The concept of blockchain technology

Blockchain is a decentralized digital ledger that ensures secure, immutable transactions without a central authority, enabling transparent governance and peer-to-peer trust (45, 46). Blockchain technology also promotes innovation and efficient resource allocation, creating opportunities for transformative solutions across multiple industries. Blockchain improves payment systems and credit information management in the financial sector, increasing efficiency and security (47). Beyond these applications, blockchain technology creates a distinct governance architecture that differs fundamentally from traditional digital platforms (3). While traditional platforms operate under what can be termed “rule by code” where platform operators maintain unilateral control over the technical infrastructure, blockchain implements a “rule of code” where rules are embedded in the technical architecture itself and apply equally to all participants (6). This governance architecture is characterized by three unique features: (1) distributed consensus that ensures all nodes produce the same order of transactions, (2) smart contracts that automate standardized interactions through predefined rules, and (3) a transparent and immutable ledger that records all transactions (4). These features enable new forms of decentralized governance that could potentially transform how sports events are managed and operated (5).

It enables secure and interoperable electronic records, fraud detection, and identity verification (48) in healthcare. It improves traceability, transparency, and efficiency in supply chain management from production to delivery (125). In the sports industry, this technology is already beginning to transform event management through non-fungible token (NFT) applications for ticketing and fan engagement, which combat ticket speculation and improve fan experiences (21). Furthermore, blockchain is also being used to protect the copyright of sporting events, ensuring secure and traceable digital rights management (40). These applications illustrate blockchain's diverse and transformative potential across sectors, emphasizing its role in increasing transparency, security, and efficiency. Furthermore, with these advancements, it is crucial to address the General Data Protection Regulation (GDPR) requirements, particularly concerning data privacy and the right to erasure, which pose challenges to the immutable nature of blockchain (49). Solutions such as incorporating off-chain data storage mechanisms or applying advanced encryption and anonymization techniques could help reconcile blockchain's inherent characteristics with GDPR's stringent privacy standards. These adaptations are essential for ensuring that blockchain applications in sports comply with legal requirements while maintaining the benefits of decentralization and transparency (25, 50).

Moreover, blockchain integrates seamlessly with emerging technologies such as big data and artificial intelligence, expanding its applications and capabilities (51). Its core properties include (a) an environment for transactions, (b) decentralized management, (c) consensus mechanisms, (d) security, (e) immutability, (f) distributed ledger technology (DLT), and (g) transparency (46, 52).

(a) *Environment for transactions*: provides a specialized environment for executing transactions and smart contracts (53).
(b) *Decentralized management*: a governance model without centralized third-party organizations, allowing autonomous operation among participants (46).
(c) *Consensus mechanisms*: mechanisms such as Proof of Work (PoW) and Proof of Stake (PoS) ensure data integrity and agreement among participants without central oversight (54).
(d) *Security*: cryptographic hash functions and digital signatures ensure the security and integrity of blockchain transactions (55).
(e) *Immutability*: data on a blockchain cannot be altered without detection, providing a secure and unchangeable ledger (56).
(f) *Distributed ledger technology*: allows data to be stored across multiple locations, enhancing security and resilience (57).
(g) *Transparency*: all participants can view transactions and data, enhancing trust and accountability within the system (58).

Blockchain integration in managing small and medium-scale sports events emphasizes its potential to enhance community engagement and organizational efficiency. These types of events serve as strategic tools for local development due to their positive economic impact and capacity to engage local communities actively (59, 60). Blockchain technology, recognized for its decentralization and transparency, addresses organizational challenges by enhancing security and knowledge management in sports event organizations (27, 61). Furthermore, the implementation of Smart Contracts and NFTs could revolutionize event management (21), enriching participant interaction and audience experience in line with governance models.

### 2.3 Small/medium events: a technological and managerial approach

Although smaller in scale and impact than mega-events, non-mega sporting events possess distinct features that can positively influence host communities. Often employed as strategic instruments for local development, these events tend to require fewer resources and are more likely to yield favourable or neutral economic results (59, 60). Factors like stakeholder cooperation, reliance on tourism, business size, promotional strategies, leadership direction, and organizational skills are key drivers of economic engagement and event success (62, 63). By leveraging event portfolios, they can also stimulate local tourism (64). Moreover, their community-focused nature fosters social cohesion and encourages partnerships with local actors, enhancing overall social benefits (65, 66).

Despite these advantages, non-mega events face organizational hurdles, including security concerns (27), undervaluation of co-hosted event outcomes (67), and persistent knowledge management difficulties (68). Technological issues, such as low standardization and professionalization (61), and complex asset performance evaluations (69), also hinder operations.

To overcome these barriers, blockchain-based governance offers innovative solutions through three mechanisms: access, control, and incentives (4). Access protocols enable stakeholder identification and decision rights allocation via transparent, immutable systems. Control mechanisms coordinate interactions between diverse actors through encrypted, traceable processes, while incentives—implemented via smart contracts—stimulate participation and value creation (5). For small and mid-sized events, these tools provide a cost-effective means to build trust and coordination without relying on costly centralized intermediaries (3). Embracing these technologies can improve organizational performance and responsiveness (61). making them especially relevant in increasingly digital event ecosystems. Table 1 summarizes how blockchain mechanisms align with key challenges, presenting associated benefits, risks, and implementation requirements.

**Table 1:** Small/medium events blockchain implementation matrix.

Dimensions	Current challenges	Blockchain solutions	Expected outcomes
Security & Trust	▪ Ticket forgery and unauthorized resale ▪ Data security issues	▪ NFT tickets with unique verification ▪ Smart contracts for automated authentication ▪ Distributed ledger for secure data storage	▪ Enhanced ticket security ▪ Improved trust by participants
Resource Management	▪ Limited resources ▪ Low professionalization	▪ Automated processes through smart contracts ▪ Decentralized resource allocation ▪ Cost-effective management solutions	▪ Reduced administrative costs ▪ Optimized operational efficiency
Stakeholder Engagement	Community participation challenges Limited fan interaction	▪ DAO-based governance ▪ NFT-enabled engagement ▪ Token-based incentives	▪ Enhanced community involvement ▪ Increased fan loyalty and participation

Adapted from Pitelis et al. (1997) (41), Teece (2007) (70), Emerson and Nabatchi (2015) (71); Helfat and Martin (2015) (126), Beck et al. (2018) (5); Werbach (2018) (3); Schmeiss et al. (2019) (4); Jun-Ming and Jing (2021) (40); Santana and Albareda (2022) (72); Mahajan et al. (2023) (13); Sombat and Ratanaworachan (2023) (73); Sung et al. (2023) (74); Glebova and Madsen (2024) (29).

As shown in Table 1, blockchain technology provides specific solutions to address the main challenges faced by small and medium-sized sports events. The chart demonstrates how blockchainenabled mechanisms can transform traditional event management challenges into opportunities for enhanced efficiency, security, and stakeholder engagement. Each dimension represents a critical area where blockchain implementation can create significant value, while also highlighting the necessary capabilities and considerations for successful implementation.

During the COVID-19 pandemic, new challenges to organizing small and medium-sized sports events were raised, encouraging sports organizations to adopt innovative strategies and technologies to ensure that such events take place (75). The significance and competitiveness during the pandemic compelled these organizations to implement novel digital technologies, including live-streaming platforms, online registration and management systems, and virtual interaction tools for participants (76). In the context of these technological adaptations, blockchain technology surfaced as a notably effective solution to further enhance event management processes (21). By harnessing blockchain, organizations can tackle various persistent challenges accentuated during the pandemic, including the assurance of security, transparency, and operational efficiency within a predominantly digital environment (77).

At this point, a fan's experience can be significantly improved by ensuring greater security, transparency, and operational efficiency (73, 78). Also, ticket management has proven especially effective in mitigating common issues such as ticket forgery and unauthorized resale (79). Using a blockchain structure for ticket management has proven especially effective in mitigating common issues such as ticket forgery and unauthorized resale (73). These NFT tickets ensure that each is unique and easily verifiable, increasing fan's trust in the authenticity of the tickets purchased (80), allowing traceability and authenticity, and preventing ticket duplication and forgery (74). Additionally, NFTs can offer additional benefits such as exclusive access to VIP areas, personalized content, and unique interactions with athletes and organizers, further enriching the participant's experience (78).

Blockchain technology has become a powerful tool for event management, significantly improving security, transparency, and efficiency (81, 82). By storing information on a distributed and immutable ledger, blockchain enables reliable tracking of transactions and activities related to events such as ticketing and credentialing. This reduces the risk of fraud and enhances trust between participants and organizers (73, 80). Additionally, blockchain-enabled smart contracts can automate essential processes such as ticket sales and participant authentication, eliminating intermediaries and optimizing operational resources (83, 84). These features are especially beneficial for small and medium-sized events where limited resources and transparency can improve engagement and security. For instance, during the “Hands-up-go” event on the Ethereum platform, organizers successfully hosted a secure and transparent experience, effectively managing participants without incurring high administrative costs. This resulted in a reliable and efficient experience for everyone involved (85).

The functionalities and applications of blockchain technologies in managing small to mediumsized sports events are extensive and offer significant contributions to enhancing the effectiveness of these events. The implementation of Smart Contracts and NFTs can revolutionize event management by introducing a transparent and participatory governance structure through DAOs (28). These technologies facilitate direct stakeholder participation in decision-making and transparent resource allocation, further promoting community-driven event development, which results in more efficient management and positively impacts both host communities and their stakeholders (52, 57). The practical application of these technologies not only enhances data security and management, but also enriches participant interaction and experience. This is supported by (59, 60) who discuss the positive economic impact and the capacity for community engagement fostered by smaller events.

## 3 Theoretical basis for DAO proposal in sports events

A way to apply blockchain technology in sports event management is by using Decentralized Autonomous Organizations (DAOs). A DAO is an organization represented by rules encoded as a transparent computer program controlled by organization members without centralized influence (24). This structure allows decentralized governance, where decisions are made democratically and transparently, using smart contracts to automate processes (23). These organizations enable participants such as fans, athletes, and sponsors to have an active voice in event decisions, promoting a more inclusive and CGT model (23). For example, governance tokens allow holders to vote on proposals and policies, ensuring that decisions reflect the community's collective will (72). DAOs can also improve financial and operational transparency as all transactions and decisions are immutably recorded on the blockchain (22). Additionally, the decentralized structure of DAOs reduces administrative costs and increases efficiency in event management (46).

Despite the growing success of DAOs in various industries, they have yet to be applied to small and medium-sized sports events. This lack of application suggests an untapped potential as DAOs could be a powerful tool for these events (52, 57). Decentralizing decision-making and fostering transparent governance enhances event management and strengthens participant engagement and loyalty, creating a more involved and committed community (71). This approach reinforces participant loyalty and builds a committed community around the event, encouraging recurring involvement and enthusiasm (86).

The theoretical foundation for the DAO model is based on the Dynamic Capabilities Framework [DCF; (41)] as its primary theoretical approach and merges domains from the CGT (43), and FMG (44). DCF provides a more suitable lens for examining blockchain technology to emphasize an organization's ability to adapt and innovate in front of technological advancements. Dynamic capabilities are defined as the firm's ability to integrate, build, and reconfigure internal and external competencies to address rapidly changing environments (87). This framework shifts focus from static, internal resources to how effectively an organization identifies (sensing), captures (seizing), and capitalizes on (transforming) new opportunities.

DCF allows organizations to transform their operations and strategic approaches in ways that sustain competitive advantages under conditions of rapid change and uncertainty (70). They emphasize the importance of possessing valuable resources, but the continual reconfiguration of these resources to maintain strategic fit with the environment (42). In the context of blockchain within sports organizations, DCF highlights how these entities can effectively sense the opportunities presented by decentralized technologies, seize these innovations by integrating them into existing processes, and transform their business models to leverage competitive advantages in a digital and dynamic landscape. The sports organizations utilizing blockchain for enhancing a better engagement or optimizing operational efficiencies must continually adapt their strategies and resources to exploit these technologies effectively (88). It is based on the premise that an organization's competitive effectiveness depends on its ability to own and exploit resources that are not easily replicable or substitutable by competitors (89). These resources can include tangible assets such as technology and infrastructure and intangible assets such as knowledge, skills, organizational culture, and relationships. The unique combination of these resources and capabilities allows organizations to develop strategies that are difficult to imitate, providing a sustainable competitive advantage (90).

**Figure 1:**
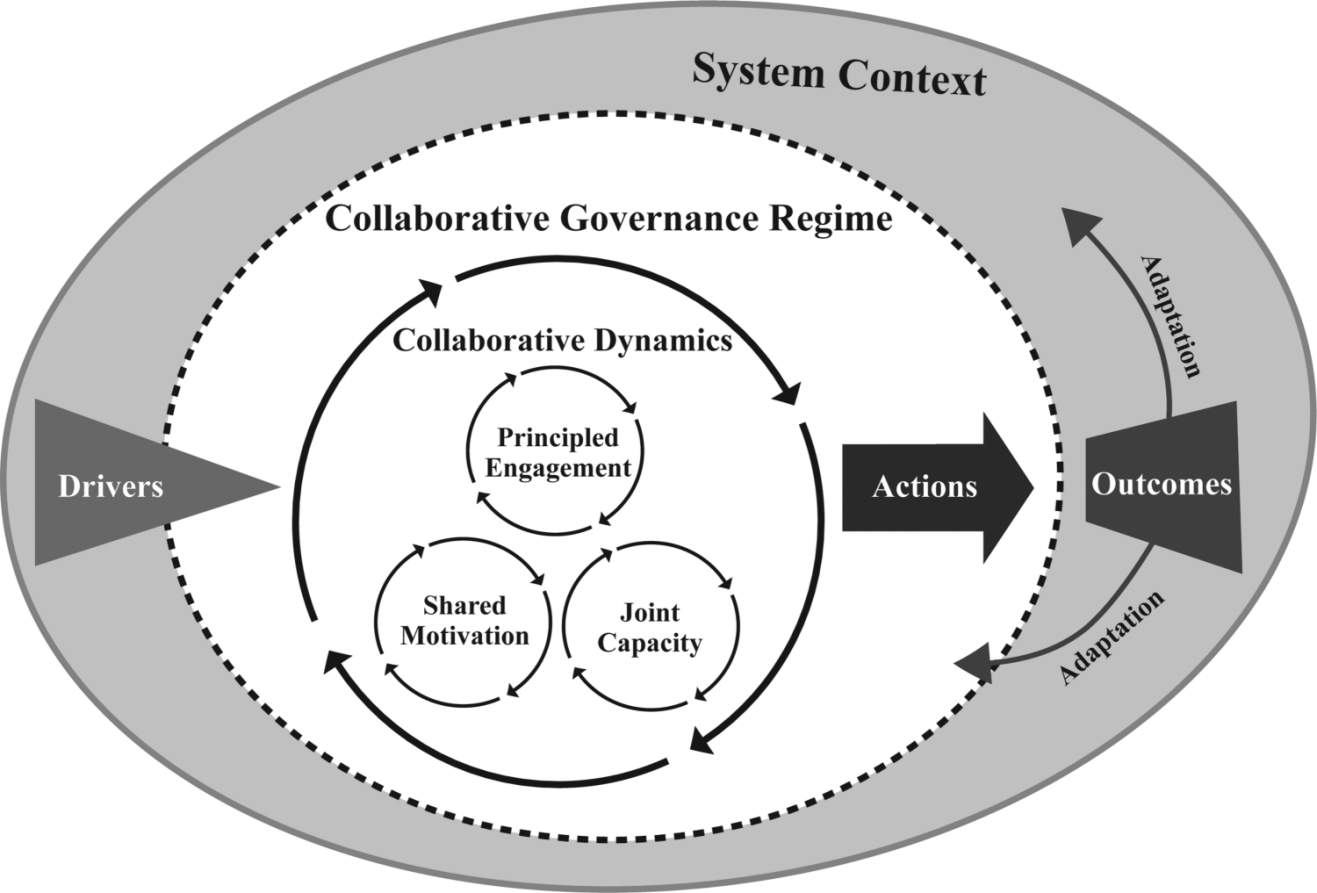
The integrative framework for collaborative governance. Adapted from Emerson and Nabatchi (2015) (71).

Complementarily, the CGT relies on three interactive components that drive the dynamics of collaboration: (1) principled engagement, (2) shared motivation, and (3) capacity for joint action (see Figure 1). Principled engagement involves a process of discovery, definition, deliberation, and determination that leads to collaborations to build a shared theory of change. Shared motivation is fuelled by perceived mutual benefits and trust, enabling actors to overcome hesitations to commit to the collaborative process and take risks for shared problem-solving. The capacity for joint action is strengthened through policies and practices that foster commitment and ensure the sustainability of collaborative governance (71). These components can facilitate the effective management of smaller-scale sports events and promote significant positive impacts for host communities and their stakeholders, integrating various stakeholders into an inclusive and transparent decision-making process.

**Figure 2:**
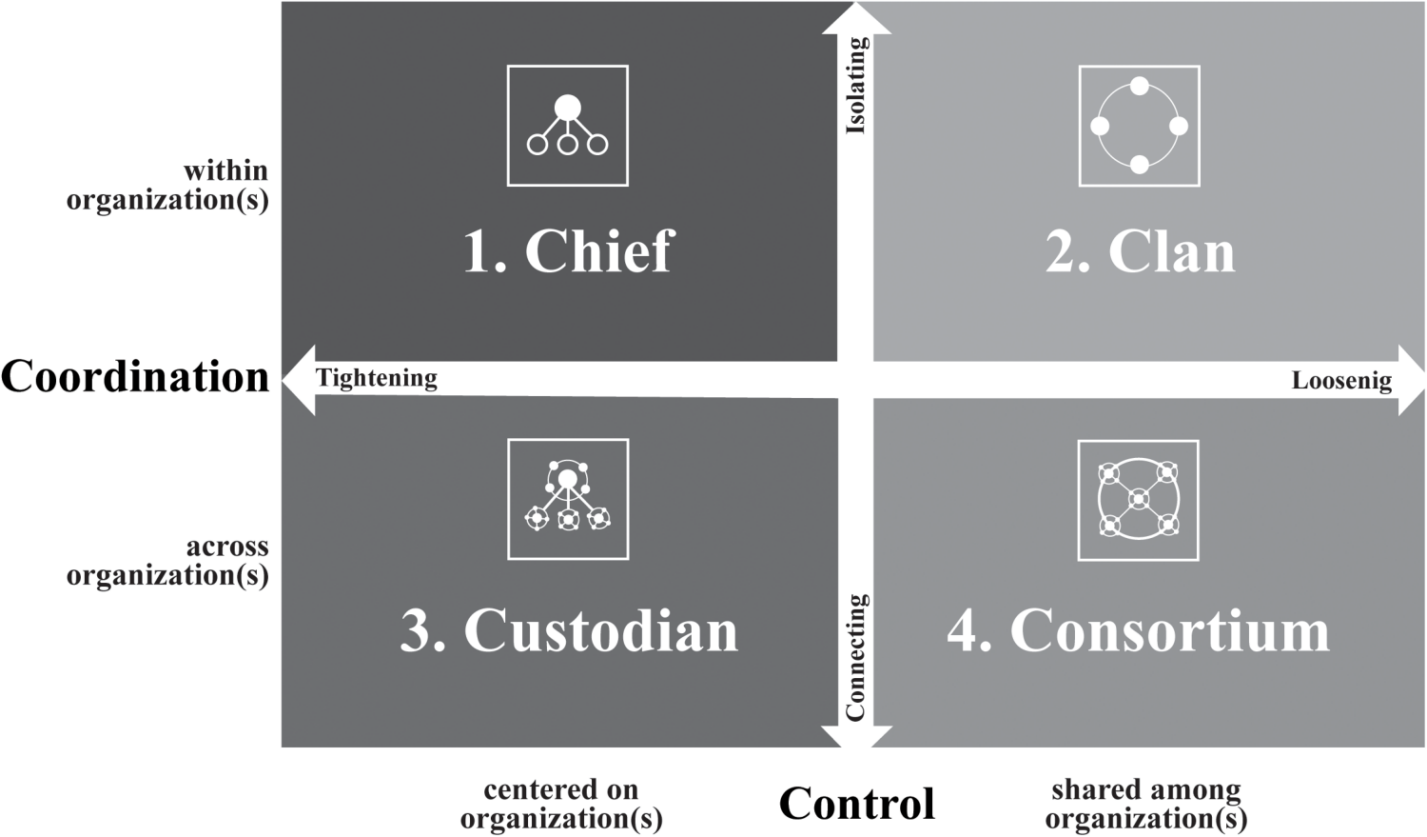
Four modes of governance for blockchains. Adapted from Goldsby and Hanisch (2022) (44).

The decentralization enabled by blockchain aligns seamlessly with the principles of engagement, shared motivation, and joint action capacity, which are fundamental in CGT (58, 71). By distributing decision-making power, blockchain reduces the concentration of authority, fostering an environment where multiple participants can effectively cooperate and coordinate their actions (43, 91). This structure enhances participation and motivates stakeholders, providing a sense of ownership and responsibility over processes and outcomes.

Moreover, CGT operates within a systemic context encompassing political, economic, and social conditions that influence collaboration (71). This context is driven by “drivers” (e.g., an influential individual, a leader, a core group, an external mediator) who catalyse the formation of the governance regime, such as the perception of significant uncertainties and the need for interdependence (92). The resulting actions lead to tangible outcomes that promote continuous learning and adaptation within the regime, allowing it to evolve in response to external and internal changes (93). Therefore, it sustains a constant cycle of improvement and innovation in collaborative governance.

Disruptive technologies such as blockchain can overcome problems by revolutionizing traditional methods and designs, significantly impacting the use of goods and services to achieve organizational objectives (58). These technologies can manage large amounts of digital data quickly and efficiently, resolving transparency and data manipulation issues. Consequently, the reliance on open network structures and multiplatform configurations has increased, paving the way for further innovations (94).

To this end, the CGT approach serves as the primary theoretical foundation, while DCF and the FMG provide structured pathways for understanding the diverse governance approaches within blockchain networks (44). This model categorizes governance into four distinct modes: (a) Chief, (b) Clan, (c) Custodian, and (d) Consortium, each offering unique strategies for managing coordination and control (see Figure 2). By applying these modes, blockchain can transform governance structures, making them more transparent, decentralized, and responsive to stakeholder needs.

An FMG framework presents multiple management strategies within blockchain networks. The “Chief mode” focuses on control in a single organization, making it ideal for environments that require quick decision-making and centralized security. The “Clan mode” supports decentralized governance within an organization, enhancing internal collaboration and innovation. The “Custodian mode” involves centralized oversight by a leading organization coordinating multiple stakeholders, ensuring stability and compliance. And the “Consortium mode” offers the highest level of decentralization with multiple organizations sharing control and responsibilities, promoting transparent and equitable decision-making, suitable for projects seeking open and inclusive governance (44).

Blending these governance modes within blockchain frameworks significantly impacts CGT by emphasizing participatory and inclusive structures. “Consortium” and “Clan” modes align with the principles of collaborative governance such as engagement, shared motivation, and joint action. These modes foster a governance environment where stakeholders are encouraged to collaborate transparently and equitably, building trust and empowering communities (44, 71).

DCF and FMG can interact complementarily to enhance governance effectiveness. By applying FMG governance modes, the strategic resources identified by DCF can be optimally leveraged. For instance, the “Chief” mode utilizes strong leadership and decision-making capabilities to maintain centralized control; this aligns with DCF's emphasis on seizing capabilities where decisive leadership can rapidly respond to market changes (44, 70). Similarly, the “Clan” mode leverages organizational culture and human capital to foster collaboration and drive innovation, reflecting DCF's focus on transforming capabilities that adapt and reshape organizational practices to sustain competitive advantage (42, 44). “Custodian” mode, which emphasizes oversight and compliance, utilizes robust procedural resources to ensure stability and efficiency across operations, aligning with DCF's sensing capabilities that monitor and assess external regulatory changes and internal performance metrics (41, 44). And, the “Consortium” harnesses strategic partnerships and mutual trust among diverse entities to promote shared governance and resource pooling, capitalizing on DCF's approach to dynamically reconfigure assets and relationships to better navigate complex business environments (44, 95).

By integrating DCF with FMG, organizations can create governance structures that are aligned with their operational needs but also agile enough to sustain competitive advantages in rapidly changing environments (87). This integration becomes particularly relevant in blockchainbased systems where governance mechanisms must address what the term “paradox of openness” (4), that is the tension between enabling maximum openness for value creation while ensuring effective value capture for all participants. The technical architecture of blockchain can help resolve this paradox through standardized interactions and automated enforcement mechanisms that protect value appropriation while maintaining system openness (5). Governance adapted from FMG, informed by dynamic capabilities, can lead to more effective decision-making processes and greater organizational resilience. Additionally, blockchain can contribute to a decentralized CGT model, offering innovative approaches to define relationships, reduce corporate risks and inefficiencies, and manage conflicts while ensuring data security and integrity in a distributed network (96). These dynamic capabilities enable organizations to not just react to environmental changes but also proactively shape their governance structures to optimize both technological and strategic outcomes.

This governance model enhances security and community empowerment by encouraging active participation in the decision-making process (97). Community empowerment is an economic development concept emphasizing societal values to build a new people-centred, participatory, and sustainable paradigm (98, 99). Blending its principles with blockchain technology promises to foster inclusive and sustainable development, enabling communities to actively participate in and benefit from technological advancements and economic opportunities (100). Moreover, it facilitates intentional interaction with evolving governance structures (principled engagement) by promoting unique and shared relationships (shared motivation) and enhancing the ability to collaborate effectively (joint capacity).

To expand comprehensively, CGT addresses individual perceptions of efficacy and involvement (71, 92) in blockchain initiatives within the sports industry. At the same time, FMG recognizes that different cultural values influence participant behaviour in these initiatives (44). Blending these theoretical premises allows us to evaluate the collaborative experience in adopting blockchain projects in various contexts. Integrating these theories helps explain contextual and organizational differences in governance approaches, discussing how these differences may arise and the underlying impacts they may generate.

Building on these theoretical foundations, creating DAOs can be argued as an effective approach to managing small-medium sports events guided by the principles of DCF, CGT and the FMG. DAOs, operating in a decentralized manner using blockchain technology enable greater participation and transparency in decision-making, reducing the concentration of power and promoting collaboration among multiple stakeholders (58, 71). This model is especially beneficial for smaller sports events where flexibility and inclusion are crucial for longterm success and sustainability.

Integrating DCF into DAOs emphasizes the development and leveraging of the capability's dynamics, such as rapid adaptation to market changes, integration of advanced technologies, and reconfiguration of organizational resources, to manage sports events effectively (101, 102). As a dynamic capability, blockchain technology provides security, transparency, and efficiency, which are essential for effective sports event management (33). Tokens from the DAOs can serve as transactional currencies within the event ecosystem and as governance tools, allowing token holders to participate in event decisions. Moreover, using Non-Fungible Tokens (NFTs) as access tickets can help event managers control ticket distribution, enhance security, and reduce fraud. This approach leverages blockchain's immutability to ensure ticket authenticity and ownership, aligning with DCF's emphasis on seizing technological opportunities to transform traditional business models and governance structures (33, 43).

The FMG governance modes can be applied flexibly within DAOs to address the unique needs of sports events. For instance, in the “Consortium”, it is possible to promote decentralized governance and equitable decision-making, particularly suitable for events seeking an inclusive and collaborative approach (44). By combining these modes with CGT principles, DAOs can create governance structures that meet their immediate needs and sustain competitive advantage over time, continuously adapting to changes and promoting innovation (71, 96). Similarly, integrating DAOs in managing small to medium-sized sports events by using VRIN resources and applying FMG governance modes offers a robust approach to enhancing efficiency, transparency, and inclusion. Through the tokens it is possible to empower the local community, allowing community members to own and trade tokens and encourage active participation and support for the events. Furthermore, NFTs can be used as tickets and digital memorabilia, creating new engagement and fan loyalty (98, 99).

Our proposed model enhances an event's adaptive and innovative capacity and promotes an environment of trust and engagement among all stakeholders, ensuring the long-term success and sustainability of a sports event (58, 71). To synthesize the theoretical foundations of our proposed framework, Table 2 presents the key concepts and contributions of each theoretical approach to synthesize the theoretical foundations of our proposed framework. When integrated with blockchain technology, this conceptual framework illustrates how DCF, CGT, and FMG provide complementary perspectives for understanding and implementing effective governance in sports events.

**Table 2:** Conceptual framework of blockchain-based sports event governance.

Theory	Key concepts	Contribution to framework
Dynamic Capabilities Framework (DCF)	Sensing Capabilities Seizing Capabilities	Technical Infrastructure Dynamic Resources Reconfiguration
Collaborative Governance Theory (CGT)	Transforming Capabilities Shared Motivation Joint Action	Stakeholder Relationships Decision-making Processes
Four Modes of Governance (FMG)	Chief Clan Custodian Consortium	Governance Structure Control Mechanisms
Blockchain Technology	Decentralization Smart Contracts Immutability	Technical Architecture Trust Mechanism

Adapted from Teece (2018) (87), Teece (2007) (70), Ansell and Gash (2008) (92), Emerson and Nabatchi (2015) (71), Helfat and Martin (2015) (126), Werbach (2018) (3), and Sato (2021) (46).

As shown in Table 2, each theoretical perspective contributes distinct but complementary elements to our framework. DCF provides the foundation for understanding how blockchain technology can be leveraged to rapidly adapt to market changes, integrate advanced technologies, and reconfigure organizational resources, thereby creating sustainable competitive advantages. CGT offers insights into how stakeholder relationships and decision-making processes can be structured in a decentralized environment to enhance transparency and participation. FMG delineates specific governance modes that can be implemented through blockchain technology, adapting to different organizational needs and contexts. These theories provide a comprehensive basis for implementing blockchain-based governance in sports events, facilitating innovation and effective management.

**Figure 3:**
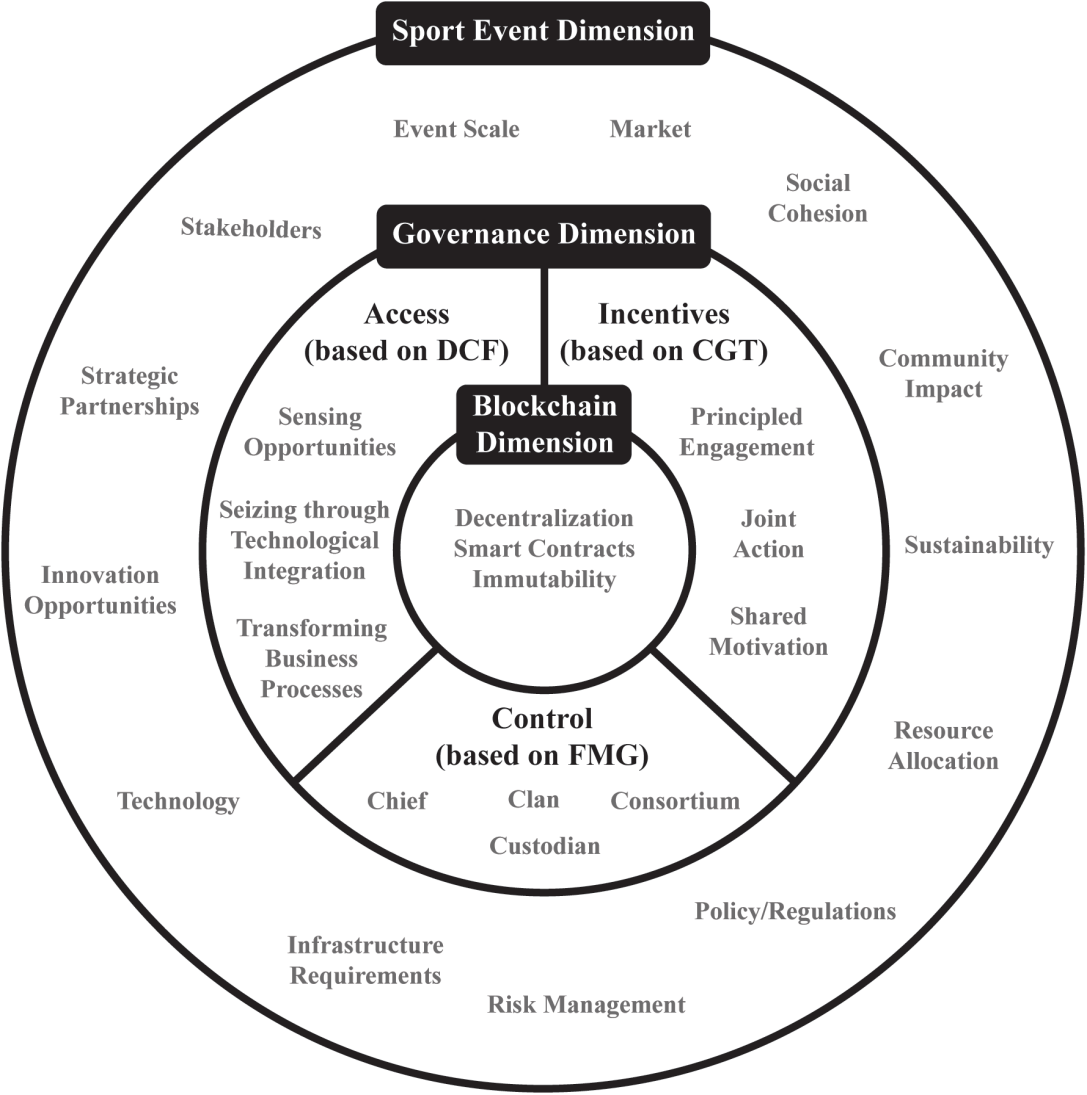
Integrated framework for blockchain-based sports event governance. Created by the authors.

Building on these theoretical foundations, Figure 3 was developed and presents an integrated framework for blockchain-based sports event governance. This framework illustrates how the multiple elements interact across three distinct but interconnected layers: the external environment, governance mechanisms, and the blockchain core.

As illustrated in Figure 3, our framework represents a dynamic system where blockchain technology serves as the core infrastructure supporting various governance mechanisms. The innermost layer comprises the essential blockchain components—smart contracts, tokens, DAOs, and NFTs—that enable automated and transparent operations. The intermediate layer represents the governance mechanisms of access, control, and incentives, each informed by our theoretical foundations: access mechanisms derived from DCF emphasizing how capabilities dynamics enable organizations to adapt and seize opportunities rapidly, control mechanisms based on FMG, and incentive mechanisms guided by CGT principles. The outer layer represents the sports event environment, including all stakeholders, regulatory requirements, market conditions, and technological developments that influence and are influenced by the governance system.

## 4 Managerial implications

Our research findings provide several practical implications for sports event managers considering blockchain implementation. The successful adoption of blockchain technology in sports events requires a strategic and phased approach to implementation. Event managers should begin with pilot projects focused on specific operational challenges such as ticketing fraud or fan engagement. This allows organizations to test and refine their blockchain implementation while minimizing potential risks and disruptions to existing operations.

From an operational perspective, managers must conduct assessments of their current technological infrastructure and identify necessary upgrades or modifications to support blockchain integration. This includes evaluating different blockchain platforms based on scalability requirements, transaction costs, smart contract capabilities, and integration possibilities with existing systems. The development of standard operating procedures and contingency plans is crucial for maintaining operational continuity during and after blockchain implementation.

Stakeholder engagement represents a critical success factor in blockchain adoption. Event managers should develop comprehensive training programs for different stakeholders, including event staff, technical teams, partners, and sponsors. When implementing DAO structures, clear governance protocols must be established, detailing voting mechanisms, decision-making processes, token distribution strategies, and participation incentives. These protocols should be designed to encourage active participation while ensuring fair and transparent governance.

Risk management considerations are paramount in blockchain implementation. Managers must identify and assess potential technical vulnerabilities, operational disruptions, regulatory compliance issues, and market acceptance challenges. Developing robust security protocols for smart contract deployment, token management, access control, and data protection is essential. This should be complemented by clear mitigation strategies for each risk category identified to ensure system resilience and stakeholder confidence.

Financial planning represents another key element of blockchain implementation. Event managers should develop comprehensive cost analyses that include initial infrastructure investment, training and development costs, operational expenses, and maintenance requirements. Additionally, new revenue opportunities through NFT-based ticketing, fan token programs, digital memorabilia, and enhanced fan experiences should be carefully evaluated. This financial assessment should consider both direct cost savings from improved operational efficiency and indirect benefits from enhanced stakeholder engagement.

Performance monitoring systems must be established to track the success of blockchain implementation. These systems should measure technical performance metrics such as transaction speed and system uptime, operational efficiency improvements, user adoption rates, and financial performance indicators. Regular assessment of these metrics enables continuous improvement and helps demonstrate the value of blockchain implementation for stakeholders. This systematic approach to performance monitoring also aids in identifying areas requiring adjustment or enhancement in the blockchain implementation strategy.

These practical implications provide sports event managers with a structured approach to blockchain implementation that balances innovation with operational stability. By following these guidelines, organizations can work toward successful blockchain adoption while minimizing risks and maximizing potential benefits for all stakeholders.

### 4.1 Technological layout for blockchain in sports event management

Integrating blockchain technology into sports event management represents a transformative and significantly improving governance, operational efficiency, and stakeholder engagement through innovative solutions for data integrity, automation, and decision-making (101, 102). Blockchain provides a secure and immutable ledger facilitating transactions, enhancing transparency, and ensuring regulatory compliance (5, 49). For example, in sports event management, blockchain-based smart contracts can automate financial transactions and enforce compliance with predefined rules, ensuring that revenue distribution, ticket sales, and sponsorship agreements remain tamper-proof and auditable in real time. This application mitigates fraudulent activities and enhances trust among stakeholders, as demonstrated by blockchain implementations in large-scale sporting events (25).

A dual-layer architecture approach is particularly suitable for sports event management because it balances security and transparency. By separating public and private blockchain layers, organizations can protect sensitive information while maintaining an open and decentralized system (103), for ticketing and fan engagement. This architecture ensures that regulatory compliance and data integrity are upheld without compromising the efficiency of automated processes in stakeholder interactions (25). A dual-layer blockchain architecture is proposed to optimize governance and security in sports event management:

• **Private Layer:** This layer securely manages sensitive personal data and financial transactions, ensuring compliance with data protection regulations such as GDPR (25, 49). Advanced technologies, including zeroknowledge proofs, homomorphic encryption, and off-chain storage mechanisms, allow selective information disclosure without compromising blockchain immutability and decentralization (46). Permissioned blockchains are also utilized, limiting access to authorized users and ensuring auditability and data integrity (104).
• **Public Layer:** This layer manages NFT-based ticketing, fan engagement, and transparent governance processes, promoting trust and decentralization and enabling secure and direct stakeholder interaction (74, 80).

In this dual-layer context, smart contracts automate sports event management, reducing manual oversight and increasing efficiency and trust (105). For instance, in 2023, FIFA implemented blockchain-based smart contracts for ticketing and access control during the World Cup, ensuring that ticket resale and validation were automated, reducing fraud and, ensuring secure transactions (106). Similarly, in professional tennis tournaments, blockchain-enabled contracts have been used to automate prize distribution among players, sponsors, and organizers, eliminating delays and discrepancies (107). Such contracts automate key processes such as ticket verification, athlete contracts, revenue distribution, and compliance monitoring, ensuring all transactions follow predefined protocols (3). These align with CGT, supporting decentralized governance models that allow multiple stakeholders to verify and enforce agreements without intermediaries (92).

In this scenario, blockchain technology facilitates inclusive and transparent governance through DAOs, enabling event managers, athletes, sponsors, and fans to participate actively in decisionmaking via token-based voting mechanisms (102). Voting mechanisms can be employed for critical decisions like venue selection, rule changes, or financial allocations, ensuring transparency (105). This DAO-based approach aligns with CGT, promoting shared authority and decentralized consensus-building (71). However, the implementation of DAOs in sports event management comes with certain limitations and prerequisites. Effective DAO adoption requires high levels of technological literacy among stakeholders, as well as clear governance structures to prevent conflicts arising from decentralized decision-making. Additionally, regulatory and legal challenges remain a key concern, as decentralized governance models may not align with existing sports governance frameworks or compliance requirements (25). Also, ensuring stakeholder engagement and participation in DAO decision-making processes is crucial to avoid centralization tendencies within decentralized systems. Addressing these challenges is essential for DAOs to function effectively in sports event management while maintaining transparency and trust (108). DCF and FMG, sports organizations can leverage blockchain to establish agile, adaptive governance structures capable of evolving with technological advancements and stakeholder needs (44, 70, 102).

DCF's sensing, seizing, and transforming capabilities enable organizations to identify emerging trends, implement smart contracts and DAOs, and continuously reconfigure governance structures for optimal performance and regulatory compliance (87). The FMG offers adaptable governance modes (hierarchical, market-based, network-based, and hybrid) that sports organizations can selectively apply to meet varying operational demands and stakeholder expectations. This flexibility ensures that governance structures remain resilient, responsive, and aligned with strategic goals amidst changing technological landscapes and evolving stakeholder relationships (44).

The blockchain's technical architecture directly contributes to risk management and fraud prevention (109). Common industry issues such as ticket scalping, counterfeit merchandise, and unauthorized access can be mitigated by blockchain solutions including NFT-based ticketing and supply chain tracking for merchandise authentication (74, 80, 82). Self-executing smart contracts also reduce financial fraud, ensuring automatic fund distribution according to predefined agreements and eliminating disputes and inefficiencies (3).

Blockchain-based decentralized identity management verifies athletes, coaches, and participants, ensuring secure credential verification processes (49). Using decentralized identifiers and verifiable credentials, sports organizations comply with privacy regulations such as GDPR, aligning with DCF's emphasis on reconfiguring digital resources (25). Additionally, fungible and non-fungible tokens engage fans in decision-making processes and exclusive experiences. NFT-based tickets provide enhanced security against fraud, ensuring authenticity and unique ownership (5). Blockchain-based automated mechanisms, such as AI-driven smart contracts, optimize real-time decision-making, adjusting policies and allocating resources without centralized (105). These capabilities align with FMG, maintaining flexibility and stakeholder trust (44).

**Figure 4:**
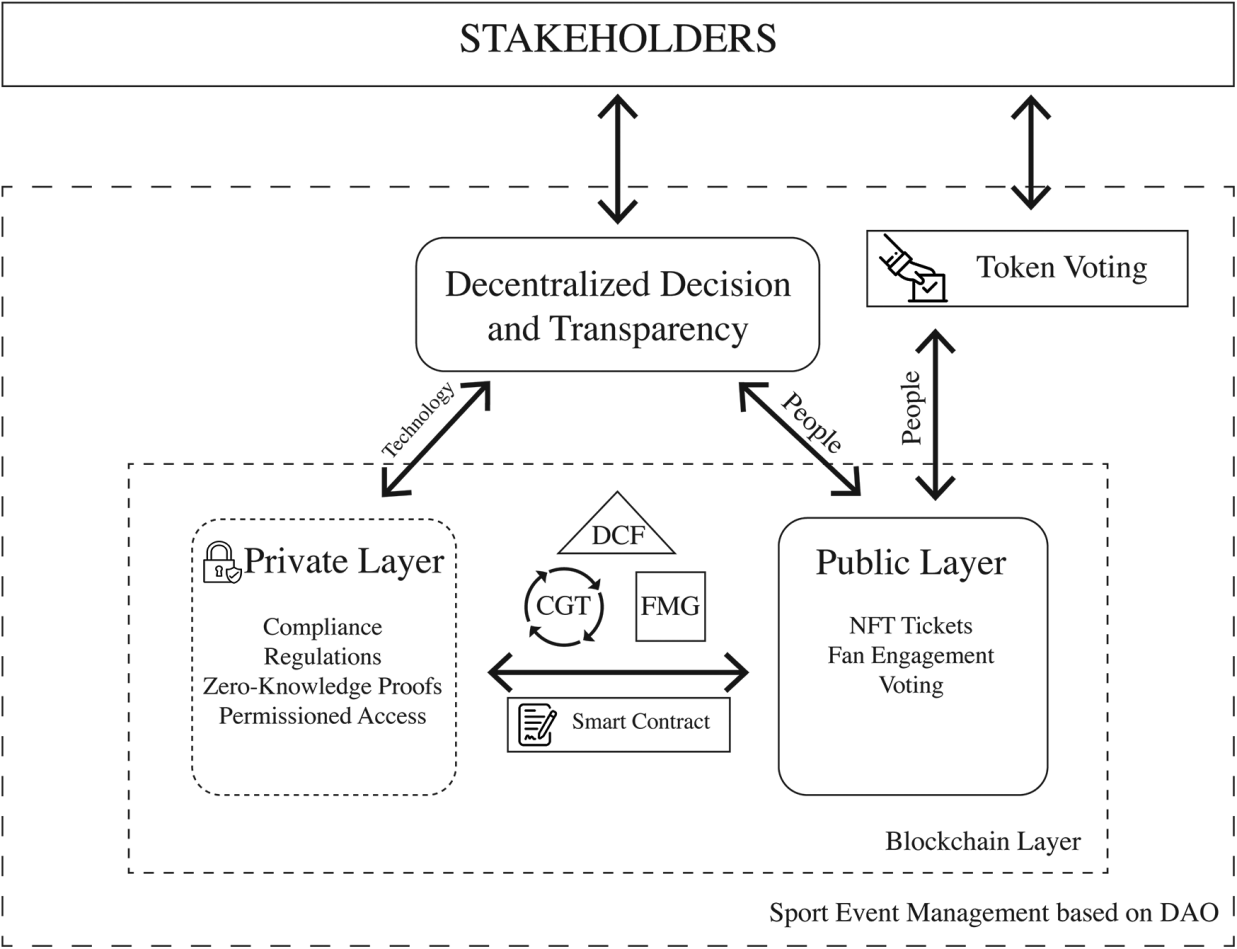
The conceptual model for sports events DAO. Created by the authors.

The evolving regulatory landscape necessitates collaborative efforts among sports organizations, technology experts, and regulators to ensure legal compliance and consumer protection (25). Thus, the blockchain integration into sports event management represents a paradigm shift driving innovation, efficiency, and inclusivity. Using DCF, CGT, and FMG frameworks, sports organizations can implement adaptive, transparent, and secure governance models, enhancing stakeholder engagement and optimizing event experiences (44, 70, 71, 102). While sport event DAOs can mitigate these challenges by providing greater transparency, inclusion, and democratization (see Figure 4). Furthermore, blockchain mechanisms can ensure that stakeholder interests are recorded and respected (102, 110, 111).

### 4.2 Empirical insights and real-world blockchain applications

With the theoretical advancements and potential applications of blockchain in sports event management, its real-world adoption emerges as a transformative approach to redefining stakeholders' engagements, management operations, and value creation (57, 112). Potential applications are diverse and impactful in sport management (113). Several use cases demonstrate the practical implementation of blockchain technology in the sports industry, providing empirical evidence that supports the proposed governance frameworks (102).

The incorporation of tokens and NFTs by sports teams has transformed the fan engagement landscape by providing distinctive digital collectibles that can be purchased, sold, and exchanged on blockchain platforms (114). Fan tokens, often issued by teams, enhance fan involvement and present unique benefits (115, 116). These tokens introduce a creative method for enhancing fan participation and support, which are critical goals for professional sports organizations. Furthermore, they have been instrumental in boosting the financial and marketing prowess of soccer clubs, showcasing their value as an economic asset and a digital marketing strategy (117). One notable example is the implementation of Fan Tokens through platforms such as Socios.com, where clubs like FC Barcelona, Paris Saint-Germain, and Manchester City allow fans to participate in decision-making through token-based voting mechanisms (106). This aligns with DAOs proposed in this study, as blockchain technology enhances transparency and fan engagement.

Another key development is NBA Top Shot, which leverages NFT-based ticketing and digital collectibles to ensure authenticity and prevent fraud (107). This supports the argument that blockchain can enhance ticket security and event access control, mitigating scalping and counterfeit ticket sales (5). Blockchain has also been explored in large-scale sporting events. The International Olympic Committee (IOC) has investigated blockchain applications for credential verification and anti-doping compliance, ensuring secure and immutable athlete records (25). Additionally, the market for blockchain in sports is projected to grow significantly, reaching $1.4 billion by 2022, with an expected annual growth rate of 8.5% until 2030 (118). The increasing adoption of NFT ticketing has led to more than 5 million blockchain-based ticket sales in 2023, reducing fraud by 80% (119). These empirical insights validate the study's proposed blockchain framework and its applicability to enhancing governance, automation, and compliance in sports event management.

Nonetheless, these innovations have not escaped criticism. They have come under scrutiny for their potential to commodify fandom and link it with gambling-like characteristics, thereby raising concerns regarding their influence on fan behavior (120). Despite these apprehensions, the introduction of fan tokens and NFTs has created new revenue streams for sports organizations, assuming an increasingly significant role within the industry (121). The use of blockchain technology in sports transformations procedures and processes within the field, while also preparing the foundation for the implementation of Decentralized Autonomous Organizations (DAOs). The DAOs is an unprecedented organizational structure in sports management with the primary feature of self-governance. Unlike the traditional vertical system of corporations, DAOs have a networked model of governance and finance (121). These organizations allow holders of tokens to convene and make major decisions automatically without any centralized control, thanks to smart contracts that automate decision-making (1, 46).

While the integration of blockchain into sports event management presents significant opportunities for enhancing transparency, governance, and stakeholder engagement, its widespread adoption still faces challenges. Issues such as regulatory uncertainties, technological literacy among stakeholders, and the volatility of digital assets must be addressed to ensure sustainable and ethical implementation. Despite these hurdles, the continuous evolution of blockchain applications, particularly through DAOs, smart contracts, and NFTs, highlights the technology's transformative potential in shaping a more decentralized and efficient sports management ecosystem. As innovation progresses, further research and real-world case studies will be crucial in refining governance frameworks and maximizing blockchain's impact on the industry.

## 5 Conclusions

This theoretical study explores the potential application of blockchain technology for managing sports events, employing the three theoretical approaches as foundational elements. The literature review indicates that although there is substantial discourse on the use of blockchain in the sports events field, particularly regarding ticket management via NFT, the practical implementation of decentralized governance models remains underdeveloped. Blockchain technology, distinguished by its robustness, immutability, transparency, and ability to automate processes through smart contracts, presents significant potential to transform sports event management. This technology offers a secure and immutable basis for transactions and records, thereby fostering a more transparent and auditable management process, which is essential for trust and verification in decentralized digital environments. Furthermore, blockchain enhances decentralized governance by empowering stakeholders to engage directly in decision-making processes, promoting inclusivity and active participation from athletes to their sport fans.

Establishing a pilot event, particularly within the eSports and exergames environment, represents a promising avenue for evaluating the effectiveness of these governance models in tandem with blockchain technology. Given the ongoing expansion of the eSports community and its inherent technological appeal, a disruptive strategy employing emerging technologies may be exceptionally well-received. This pilot initiative would facilitate not only the assessment of governance models and technologies in a controlled setting, but also the measurement of impacts—albeit on a micro scale— within the participating community. This reformation of governance models must carefully consider the design of blockchain-enabled governance mechanisms that address three fundamental dimensions: access, control, and incentives. The successful implementation of these mechanisms requires careful consideration of both technical architecture and social dynamics, balancing the need for automation and decentralization with the requirement for human oversight and intervention in exceptional circumstances. Moreover, the adoption of blockchain technologies and DAOs within the sports industry raises significant regulatory and legal challenges. The ambiguous legal status of DAOs, coupled with the decentralized nature of blockchain, complicates compliance with existing national and international sports regulations. For example, adopting decentralized finance (DeFi) models in sports organizations through DAOs requires careful navigation of financial regulations to ensure transparency and protect stakeholder interests.

To realize the potential of blockchain technology in sports management, it is imperative to foster collaborative efforts among technologists, regulators, and sports administrators. Developing regulatory frameworks that not only encourage innovation but also ensure compliance and protect stakeholder interests is crucial. These collaborations are essential to navigate in complex legal landscapes and design systems that are innovative and accountable. Working together, the stakeholders can create a beneficial environment that supports technological advancements and regulatory compliance, thus ensuring that the deployment of blockchain in sports is effective and sustainable.

Additionally, that integration of sports events must navigate the complexities of data protection regulations, for example, the GDPR in the European Union. The GDPR's stringent requirements on data privacy, including the rights to erasure and data portability, present challenges to the immutable nature of blockchain records. To address these legal constraints, it is essential to develop innovative solutions that can reconcile the benefits of blockchain's transparency and security with the need for compliance with GDPR. Strategies such as employing pseudonymization techniques and ensuring that sensitive data is stored off-chain could be crucial in aligning blockchain deployments with GDPR mandates. These measures will not only facilitate compliance but also enhance trust among participants by safeguarding personal data against misuse.

Our conceptual approach serves to inspire researchers and event managers within the blockchain domain as a legitimate instrument for reforming governance models in sports events. Further research and practical implementation should focus on developing the term “regulation via governance” rather than “regulation by code” approaches, emphasizing the importance of adaptive and responsive governance frameworks that can evolve with changing stakeholder needs, while technological capabilities will be essential in addressing existing challenges and fully harnessing the innovative potential of blockchain within the sports industry.

## 6 Limitations and suggestions for future research

This study's theoretical exploration of blockchain technology in sports events management, while contributing to the academic discourse, presents several noteworthy limitations that warrant careful consideration and suggest promising avenues for future research. A major limitation lies in the absence of empirical and anecdotical evidence to substantiate the effectiveness of blockchain technology, particularly in the context of sports events managed by DAOs. Despite the theoretical potential of DAOs to enhance event management, their practical application in sports remains largely unexplored, necessitating future empirical studies to validate the theoretical models proposed. Validation may come from case studies or pilot projects applying blockchain and DAOs, offering insights into their benefits and limitations.

While valuable in theory, existing governance frameworks face significant practical implementation challenges when applied to blockchain-based management in sports organizations. Although the Dynamic Capabilities Framework (DCF) emphasizes adaptability, its application is hindered by sports clubs' limited flexibility and digital infrastructure. Similarly, Collaborative Governance Theory (CGT), while effective in analyzing multi-stakeholder collaboration, does not easily translate into the reality of sports organizations, where traditional hierarchical structures dominate decision-making, and power imbalances persist. The lack of structured enforcement mechanisms further complicates the application of blockchain-driven governance models, making it difficult for clubs to integrate decentralized participation effectively. Likewise, the Four Modes of Governance (FMG), despite providing a clear categorization of governance structures, faces practical obstacles in adoption—many sports clubs and federations struggle with shifting from centralized control to a more decentralized, flexible approach. These challenges are particularly evident in small to medium-sized sports organizations, where financial constraints, technological barriers, and resistance to change make the transition from traditional governance to blockchain-based models difficult to operationalize.

To address these limitations, future research should focus on enhancing DCF with blockchainspecific dynamic governance models that explicitly account for the self-executing nature of smart contracts, tokenized governance, and decentralized decision-making structures. Additionally, developing standardized metrics for blockchain governance efficiency—including measures of trust, transparency, stakeholder engagement, and decentralization effectiveness—would improve the empirical application of governance theories in blockchain-driven ecosystems. Finally, hybrid governance models, integrating traditional decision-making principles with decentralized blockchain governance, could provide a scalable and adaptable framework for sports event management, ensuring efficiency, inclusivity, and regulatory compliance in both centralized and decentralized governance environments.

Another limitation of this conceptual study lies in its limited exploration of regulatory and legal challenges associated with implementing blockchain technologies and DAOs in sports management. Future studies should focus on developing comprehensive frameworks that address these regulatory and legal aspects, systematically identifying and analysing existing regulations that impact blockchain deployment in sports, both nationally and internationally.

The study could benefit from incorporating additional theoretical perspectives to complement the current framework. Institutional Theory (122) could be applied to examine how institutional norms and structures influence blockchain technology adoption in sports events, while the Diffusion of Innovations Theory (123) would be valuable for analysing how blockchain and DAOs are disseminated among organizers, sponsors, and local communities. Furthermore, the Innovation Ecosystems Theory (124) could provide insights into how blockchain can be integrated into broader technological ecosystems, fostering collaboration among stakeholders and generating collective value. These additional theoretical perspectives could offer a more comprehensive understanding of the sociotechnical and interinstitutional dynamics involved in implementing disruptive technologies in the sports field.
